# A Rare Complication of Rhabdomyolysis: Peripheral Neuropathy

**DOI:** 10.7759/cureus.14202

**Published:** 2021-03-31

**Authors:** Chidinma Ejikeme, Ramez Alyacoub, Sherif Elkattawy, Tanya Shankar, Ruhin Yuridullah

**Affiliations:** 1 Internal Medicine, Rutgers New Jersey Medical School/Trinitas Regional Medical Center, Elizabeth, USA; 2 Internal Medicine, St. Joseph's Univeristy Medical Center, Paterson, USA

**Keywords:** rhabdomyolysis, peripheral neuropathy, cpk

## Abstract

Rhabdomyolysis is a complex medical condition characterized by muscle necrosis and the release of intracellular components into the circulation. Although its most common cause is a direct traumatic injury, it can result from non-traumatic factors as well, including infection, toxins, and drugs. Serum creatine phosphokinase (CPK) levels are usually elevated in this condition and they correlate with the severity of the muscle damage (the higher the CPK peak, the greater the magnitude of muscle damage), although lower levels of CPK do not necessarily rule it out. The common complications associated with rhabdomyolysis include acute kidney injury, compartment syndrome, and, in rare cases, peripheral neuropathy. In this report, we present a case of a young patient, with a history of alcohol abuse, who presented with bilateral numbness of the feet post-immobilization and was subsequently found to have severe rhabdomyolysis.

## Introduction

Rhabdomyolysis is a syndrome caused by muscle necrosis and the release of intracellular muscle components, especially creatinine phosphokinase (CPK) and electrolytes, into the bloodstream [1-3]. Historically, the incidence of myopathic events and rhabdomyolysis has been challenging to evaluate in clinical research due to a lack of formal clinical definitions. The injury is mainly mediated by high intracellular calcium load and proteases, which disrupt the membrane integrity. The classic clinical presentation triad is myalgia, weakness, and tea-colored urine, although this triad may not be seen in all cases [1]. Common complications include fluid and electrolyte imbalances such as metabolic acidosis, hyperkalemia, hyperphosphatemia, hypocalcemia, acute renal failure, compartment syndrome, and disseminated intravascular coagulation (DIC) [1]. Peripheral nerve injury is rare in patients with rhabdomyolysis. Several case reports on neuropathy in rhabdomyolysis have described the development of neuropathy after prolonged immobilization due to toxin-induced coma [4,5]. In this report, we present the case of a patient who developed neuropathy as a complication from rhabdomyolysis.

## Case presentation

A 32-year-old male with a history of polysubstance abuse presented to the emergency department (ED) with complaints of bilateral feet numbness. The patient stated that he had consumed alcohol the evening prior to the presentation, and upon awakening the next morning, he had been unable to bear weight on lower extremities. He admitted to decreased sensation bilaterally, which extended from the foot to the ankle, more pronounced on the right side. Furthermore, the patient had a 7/10 dull pain sensation radiating from the lower back to bilateral lower extremities as well as generalized body aches. It was the progressive worsening of his symptoms that had prompted his hospital visit. He denied any other aggravating factors.

In the ED, the patient's vital signs were found to be remarkable for a temperature of 97.1 °F, respiratory rate of 19 breaths per minute, heart rate of 124 beats per minute, and blood pressure of 146/27 mmHg. The patient denied any history of diabetes, kidney disease, or back pain. He had smoked half pack of cigarettes a day for "a few years", drank a pint of vodka three to four times a week, and used cocaine as well as marijuana daily for "numerous" years. He reported that he had used cocaine and marijuana a "few" days prior to the presentation. The patient's physical examination was significant for a decreased dull and sharp sensation of bilateral feet with intact sensation above the ankle as well as 1/5 muscle strength of bilateral legs and feet. The muscle strength was 4/5 in bilateral thighs as well as upper extremities. 

An alcohol level screening was done in the ED, which revealed a level of less than 10 mg/dl. Urine drug screen was positive for cocaine, cannabinoids, and opiates. Lactic acid was elevated at 4.2. Urinalysis revealed a specific gravity greater than 1.030 with large bilirubin, proteinuria, and hematuria. Urine microscopy revealed red blood cells of 4-6 high power field (HPF).

**Figure 1:**
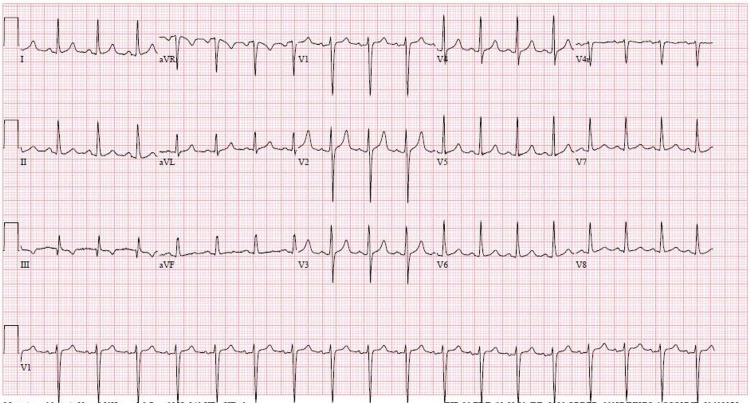
EKG remarkable for sinus tachycardia with peaked T waves in anterolateral leads EKG: electrocardiogram

Initial complete blood count revealed a WBC of 20.9 K/UL with 83.2% absolute neutrophils, and hemoglobin and hematocrit level of 21.7 gm/dl and 64.1%, respectively, with a platelet count of 344 K. The patient’s complete metabolic panel was significant for elevated liver enzymes - aspartate aminotransferase (AST): 3,334 U/L (normal range: 15-41 U/L), alanine aminotransferase (ALT): 850 U/L (normal range: 17-63 U/L), alkaline phosphatase (ALP): 151 U/L (normal range: 38-126 U/L); there was renal insufficiency with a creatinine level of 4.16 mg/dl (normal range: 0.7-1.2 mg/dl), urine nitrogen of 32 mg/dl (normal range: 8-20 mg/dl), and potassium of 7.4 mmol/l (normal range: 3.6-5.1 mmol/L) with high anion gap of 21. An arterial blood gas revealed PH of 7.23, PCO_2_ of 32 mmHg, HCO_3_ of 14.9 mmol/L, signifying metabolic acidosis with adequate respiratory compensation. CPK levels were markedly elevated at 100,809 U/L. Initial ECG revealed sinus tachycardia with peaked T wave as seen in Figure 1.

The patient received 2 grams of calcium gluconate infusion for cardiac membrane stabilization. He also received insulin and Kayexalate, which corrected his hyperkalemia. The patient was started on IV fluid (IVF) hydration with strict urine output monitoring. Initial MRI without contrast of spine was performed and was only remarkable for mild spondylosis without spondylolisthesis in the L4-L5 region. Neurology was consulted for a more thorough neurological examination, and the patient was transferred to the intensive care unit (ICU) for close monitoring and management.

In the ICU, the patient’s symptoms started to worsen and he reported progression of the numbness up to the knee in both lower limbs. He also complained of worsening pain and weakness in the right lower extremity. During the ICU stay, the right thigh became more swollen compared to the left. Urgent CT scan of the right lower extremity revealed right leg edema without focal tissue or osseous abnormality, as seen in Video 1.

**Video 1:** CT scan of the right lower extremity revealed right leg edema without focal tissue or osseous abnormality CT: computed tomography

Doppler ultrasound showed normal arterial flow and no evidence of deep vein thrombosis. The patient started experiencing new-onset right lower extremity weakness. Dorsal pedis and tibial pedis pulses were present bilaterally. At this juncture, there were concerns for acute compartment syndrome. The patient was placed on neurovascular checks every hour. Intracompartmental pressures were measured frequently, which remained low. Also, surgery was consulted for possible fasciotomy.

Despite aggressive IVF hydration, the patient’s CPK levels continued to rise. CPK levels peaked at 237,025U/L, even though his hemoconcentration had resolved. Creatinine levels continued to increase, and the patient became anuric and IVF hydration was discontinued. Vascular surgery and nephrology were consulted for emergent Uldall catheter placement and urgent hemodialysis, respectively.

Following repeat sessions of hemodialysis, the patient's renal function improved and CPK levels declined. His lower extremity weakness also started to improve. There was a significant improvement in his left lower extremity numbness. The right leg swelling resolved; however, there was only a mild improvement of motor and sensory loss. The patient continued to undergo physical therapy during his hospitalization. He was subsequently discharged to subacute rehabilitation. The patient was advised to continue with outpatient hemodialysis till full recovery of his renal function was achieved. He was also recommended to follow up with neurology as an outpatient.

## Discussion

Rhabdomyolysis is a clinical syndrome characterized by muscle necrosis and the release of intracellular components into the circulation. The causes of rhabdomyolysis can be broadly divided into traumatic or muscle compression (e.g., crush syndrome or prolonged immobilization) vs. nontraumatic. Nontraumatic causes can be further divided into exertional (e.g., marked exertion in untrained individuals, hyperthermia, or metabolic myopathies) and non-exertional (e.g., drugs or toxins, infections, or electrolyte disorders) [1]. Despite the broad spectrum of causes, they all lead to a final common pathway of ATP depletion and increased intracellular calcium, which leads to the activation of protease enzymes and mitochondrial dysfunction culminating in myocytes' death.

Clinical manifestations of rhabdomyolysis may include muscle pain, decreased muscle strength, or tea-colored urine. The diagnosis of rhabdomyolysis in a patient can be made clinically based on the presence of either an acute neuromuscular illness or dark urine without other symptoms, plus a marked acute elevation in serum creatine kinase [2]. Other manifestations of rhabdomyolysis include fluid and electrolyte abnormalities. Later complications include acute kidney injury, compartment syndrome, and, in rare cases, DIC. Acute compartment syndrome is a potential complication of severe rhabdomyolysis. It can occur in any distinct anatomic compartment bound by unyielding fascial membranes.

Peripheral neuropathy is a rare but reported complication of severe rhabdomyolysis [3]. Several mechanisms have been proposed for the peripheral nerve damage that occurs following rhabdomyolysis. The most prominent among them is compressive nerve injury, which can occur after prolonged lying or sitting. It can also happen due to direct compression of edematous muscles in a single muscle compartment [4]. In addition, compartment syndrome, which is a known complication of rhabdomyolysis, results in increased intramuscular pressure, thereby inducing compressive neuropathy. Another proposed mechanism is the spread of local inflammation and ischemia of adjacent peripheral nerves as a consequence of muscle damage [5,6]. The latter mechanism has been supported by multiple reports of cases of neuropathy without the presence of compartment syndrome and the occurrence of neuropathy outside the involved muscle compartment and not explained by the first mechanism [4-6].

Metabolic changes associated with long-term alcohol consumption may also increase the risk of neuropathy in patients with rhabdomyolysis. Alcohol itself increases the risk of rhabdomyolysis due to direct toxic effects on muscles and secondary metabolic changes associated with alcohol abuse. Ethanol consumption can alter the resting trans-membrane potential of skeletal muscle fibers and cause the disruption of adenosine triphosphatase pump function and breakdown of the muscle membrane [3,7]. Furthermore, alcohol also has direct and indirect toxic effects on neurons, which can worsen neuropathy. Alcohol metabolism results in toxic metabolites directly causing axonal degeneration and demyelination [8]. Alcohol also causes nutritional deficiencies, including thiamine deficiency.

Several factors have been suggested as predictors of the development of neuropathy in patients with rhabdomyolysis. They include marked elevation of CPK levels, dense uptake on bone scans, and abnormal MRI findings, specifically multiple muscle involvement within a compartment [4]. Since higher CPK levels, dense uptake on bone scans, and abnormal muscle intensity on imaging usually indicate severe rhabdomyolysis, these findings suggest that the risk of peripheral neuropathy is increased in severe rhabdomyolysis [6].

Electromyography and nerve conduction studies can be used to confirm peripheral neuropathy. Very little is known about the prognosis and recovery of neuropathy in patients with rhabdomyolysis [6]. Even though no nerve conduction or electromyography study was performed in our patient to confirm peripheral neuropathy, the diagnosis was made based on clinical examination with the improvement of neuropathy coinciding with decreasing CPK levels. Early recognition of rhabdomyolysis and prompt management by fluids and by correcting electrolytes and metabolic abnormalities is essential, followed by physical therapy and rehabilitation. The development of rhabdomyolysis in our patient was likely multifactorial in the setting of alcohol and drug abuse in addition to prolonged immobilization. His severe rhabdomyolysis resulted in peripheral neuropathy despite the absence of compartment syndrome.

## Conclusions

Rhabdomyolysis is a relatively common syndrome that results in a variety of complications. In this report, we highlighted one of its rare complications, peripheral neuropathy. We urge providers to appropriately use the clinical data available when evaluating patients and come up with an encompassing diagnosis.
